# Microsatellite-based phylogeny of Indian domestic goats

**DOI:** 10.1186/1471-2156-9-11

**Published:** 2008-01-28

**Authors:** Pramod K Rout, Manjunath B Joshi, Ajoy Mandal, D Laloe, Lalji Singh, Kumarasamy Thangaraj

**Affiliations:** 1 Central Institute for Research on goats, Makhdoom, Farah, Mathura-281122, India; 2 Centre for cellular and Molecular biology, Uppal Road, Hyderabad-500007, India; 3 Station de Génétique Quantitative et Appliquée, INRA, 78352 Jouy-en-Josas, France INRA, Cedex, France

## Abstract

**Background:**

The domestic goat is one of the important livestock species of India. In the present study we assess genetic diversity of Indian goats using 17 microsatellite markers. Breeds were sampled from their natural habitat, covering different agroclimatic zones.

**Results:**

The mean number of alleles per locus (NA) ranged from 8.1 in Barbari to 9.7 in Jakhrana goats. The mean expected heterozygosity (He) ranged from 0.739 in Barbari to 0.783 in Jakhrana goats. Deviations from Hardy-Weinberg Equilibrium (HWE) were statistically significant (P < 0.05) for 5 loci breed combinations. The D_A _measure of genetic distance between pairs of breeds indicated that the lowest distance was between Marwari and Sirohi (0.135). The highest distance was between Pashmina and Black Bengal. An analysis of molecular variance indicated that 6.59% of variance exists among the Indian goat breeds. Both a phylogenetic tree and Principal Component Analysis showed the distribution of breeds in two major clusters with respect to their geographic distribution.

**Conclusion:**

Our study concludes that Indian goat populations can be classified into distinct genetic groups or breeds based on the microsatellites as well as mtDNA information.

## Background

**Figure 1:**
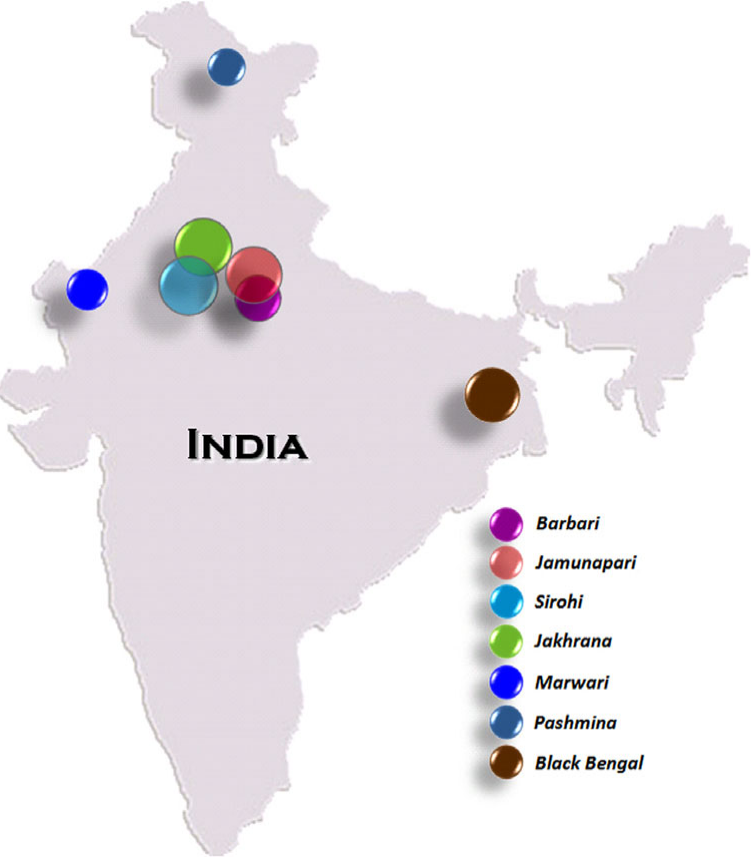
Geographical distribution of Indian goat breeds studied.

Goats, like other livestock species, are recognised as important components of world diversity and may play a useful role in sustainable agriculture in future. The goats in India are distributed in all climatic regions and evolved in isolation for a long period due to varying selective pressures and genetic drift. Goat breeds are mainly defined by their geographical position, morphological characteristics and production performance. Goats in India can also be divided into large, medium and small breeds, which are mainly distributed into four major geographical regions: temperate Himalayan region, northwestern region, southern peninsular region and eastern region [1]. Goat breeds have also been classified based on their production status: milk producing, meat producing and dual type. In addition to this, considerable variation exists among goat populations in terms of size, coat colour, ear, horn pattern and production performance. In the present study we have included all major goat breeds of India, representing diverse production characteristics (Fig 1 and Table 1). The Jamunapari breed is known as the best Indian dairy goat [2] and is found in isolated pockets in the Chakarnagar area of the Etawah district of Uttar Pradesh (UP). The Jakhrana, also known for high milk yield, is from the Alwar area of Rajasthan. The Barbari is a medium size dual-purpose breed known for its adaptability over a wide range agro-climatic situation. The Sirohi is a medium to large size breed and is used for both meat and milk in its natural habitat. The Marwari is a medium size breed and is known for hardiness and adaptability to extreme temperature. The Black Bengal is the typical dwarf breed of eastern India and known for high prolificacy and meat quality. Pashmina goats are distributed in high altitude of Himalayan region and known for the best fibre production. In addition to the major breeds, there are many intermediate and less distinguishable goat subtypes; however the genetic relationship among the major types as well as subtypes has not been established.

**Table 1:** Summary of goat breeds sampled

**Breed**	**Native tract**	**Utility**	**DNA sample**
Barbari	Mathura, Agra (UP)	Medium size, known for both milk and meat	37
Jamunapari	Chakarnagar, Etawah (UP)	Large size known for milk production	49
Black Bengal	Kolkata (WB)	Small size breed known for meat and skin quality	49
Pashmina	Ladakh	Best fibre producing breed	31
Jakhrana	Jhakarana, Behror, alwar (Rajsthan)	Large size known for milk production	50
Marwari	Desh-Nokh, Bikaneri (Rajsthan)	Large size breed known for meat, milk and coarse fibre	35
Sirohi	Tonk, Udaipur (Rajsthan)	Large size known for meat and milk	51

An assessment of genetic variability in domestic goats is a first step towards conservation of genetic resources for maintaining breeding options. In the changing phase of agricultural practices, a few breeds have been used on a large scale for immediate economic gain. Therefore, locally adapted native breeds have been neglected or displaced without knowing their genetic importance. DNA markers have been used to study the genetic variation in livestock, human and other populations [3-5]. Genetic markers are used to determine genetic variation between breeds; subsequently relationships among breeds are determined in calculating genetic distance and constructing trees. Microsatellite markers have been used as good tools to analyse the genetic variation in cattle, sheep, pig, and goats [6-11]. Using the maternally inherited mitochondrial DNA (mtDNA) sequence information, we have demonstrated earlier the existence of all the three previously known lineages A, B, C and proposed two new lineages among Indian goat populations [12]. However, no study has been conducted on Indian goats using the nuclear microsaltellite/short tandem repeats (STR) loci and covering a wide set of populations from different agroclimatic zones on Indian goats. Therefore, we have characterized seven economically important Indian goat breeds using STR markers.

## Results

### Genetic variation in Indian goats

Out of 23 STR markers analysed, 4 failed to amplify in any of the samples, 2 showed monomorphic patterns and the remaining 17 were polymorphic. The total number of alleles and allele size range for each locus are presented in Table 2. Among the polymorphic markers, the BM4621 and SRCRSP9 loci showed the highest number of alleles (more than 20) in the populations analyzed. The IDVGA7, BM6526, INRABERN192, TGLA40 and SRCRSP5 loci showed more than 15 alleles. The remaining 10 loci showed less than 15 alleles. Locus SRCRSP10 exhibited the smallest number of alleles (9). The total number of alleles varied from 9 (SRCRSP10) to 22 (BM4621). In total, 248 alleles were observed from 17 loci surveyed.

**Table 2:** STR markers its localization, allele range along with annealing temperature

**Marker**	**Chromosome number**	**Number of alleles identified**	**Allele range(bp)**	**Heterozygosity**	**Annealing temperature**
BM4621	6	22	106–148	0.786	58° for 30 sec
NRAMP	2	13	220–248	0.785	55° for 15 sec
Oar AE101	6	13	90–120	0.807	54° for 20 sec
IDVGA7	25	16	210–240	0.765	60° for 30 sec
ILST S005	10	12	172–198	0.597	61° for 15 sec
BM6526	27	17	142–178	0.805	58° for 45 sec
ETH 225	14	12	138–160	0.733	58° f or 20 sec
Oar HH 56	23	14	150–178	0.796	63° for 15 sec
INRABERN92	7	18	152–208	0.752	55° for 30 secs
Oar FCB48	17	12	150–192	0.751	55° for 30 secs
Oar HH62	20	12	108–130	0.713	58° for 45 secs
TGLA 40	-	16	170–200	0.725	57° for 30 secs
BM 143	6	13	96–120	0.709	50° for 30 sec
SRCRSP 5	21	17	150–186	0.800	55° for 15 sec
SRCRSP6	19	13	130–160	0.732	55° for 15 sec
SRCRSP 9	-	21	112–156	0.858	60° for 30 secs
SRCRSP 10	8	9	260–276	0.755	55° for 15 sec

### Genetic variation within breeds

The most polymorphism was detected at the SRCRSP9 locus (0.85) and the least polymorphism at the ILST S005 locus (0.597). Breed specific alleles were observed at different loci for different breeds with low frequency. Measures of genetic variability are shown in Table 3. Average observed and expected heterozygosity ranged from 0.375 to 0.426 and 0.739 to 0.783, respectively. The mean expected heterozygosity (He) ranged from 0.739 in Barbari to 0.783 in the Jakhrana breed. The Barbari showed the lowest gene diversity, while the Jakhrana and Sirohi showed the highest gene diversity among Indian goats. Wilcoxon's signed ranks test indicated that He was significantly lower (P < 0.05) in Barbari goats than in other breeds.

**Table 3:** Measures of genetic variability in Indian goats

**Breed**	**Sample size**	**Total number of alleles (TNA)**	**Alleles/locus (NA)**	Corrected Alleles/locus*	**Heterozygosity (Observed)(H_O_)**	**Heterozygosity (Expected)(H_E_)**	**HWE deviation**
Barbari	37	140	8.1	7.8	0.384	0.739	1
Jamunapari	49	153	9.0	8.2	0.381	0.769	1
Black Bengal	49	151	8.9	8.5	0.384	0.776	1
Jakhrana	50	165	9.7	8.9	0.387	0.783	0
Marwari	35	148	8.7	8.6	0.426	0.774	1
Sirohi	51	162	9.3	8.6	0.386	0.781	0
Pashmina	31	129	7.6	7.6	0.375	0.760	1

The mean number of alleles per locus (NA) varied from 8.1 in Barbari to 9.7 in Jakhrana goats. The mean number of alleles per locus (NA) corrected for sample size (calculated based on n = 31) is presented in Table 3. The comparison between both estimates was different in some breeds due to variation in sample size. The most diverse goat breeds were the Jakhrana and Sirohi, which had the highest total number of alleles (TNA) of 165 and 162 and highest mean number of alleles (MNA) of 9.7 and 9.3, respectively. The least diverse breed was the Pashmina, which had the lowest TNA of 129 and the lowest MNA of 7.6. Average and expected heterozygosity was lowest in the Pashmina. Similarly the Jakhrana and Sirohi had the highest expected heterozygosity of 0.783 and 0.782, respectively. However, the Marwari had higher observed heterozygosity than the Jakhrana and Sirohi. Deviations from Hardy-Weinberg Equilibrium (HWE) were statistically significant (P < 0.05) for 5 loci breed combinations. These loci included one each in Barbari (ILSTS 005), Jamunapari (ILSTS 005), Black Bengal (SRCRSP10), and Pashmina (Oar HH56) and Marwari (ETH 225). However, the total number of significant deviations was below the 5% level in each population.

F_ST _values for each pair of populations varied from 0.036 to 0.088. The average G_ST _values over all loci was 0.080, indicating that a 8.0% of total genetic variation corresponded to differences among populations, whereas 92.0% was explained by difference among individuals. The average R_ST _value (based on the stepwise mutation model) over the loci was 0.177. Mean pairwise comparisons between breeds showed that R_ST _values were 2–4 folds higher than G_ST _values. An exact test for population differentiation for all pairs of breeds across all loci showed that all breeds were significantly (P < 0.001) different from each other.

Further, an AMOVA analysis was carried out to analyze the variation within and between breeds. The AMOVA revealed that percentage of variation among populations was 6.59% and within populations was 93.41%. Variance components among population were highly significant for all the studied loci (Table 4), demonstrating significant geographical structuring in Indian goats. ILSTS005 and BM4621 contributed 14.42% and 11.50% variability among populations, respectively, and SRCRSP6 and NRAMP showed the lowest variability among populations (2.26% and 3.28%, respectively).

**Table 4:** AMOVA analysis of Indian goat breeds based on microsatellite DNA variation

**Source of variation**	**Degree of freedom**	**Sum of squares**	**Variance component**	**Percentage of variation**
Among Populations	7	294.747	0.466	6.59
Within populations	610	4034.047	6.613	93.41

**Total**	617	4328.794	7.080	

### Genetic distance

**Figure 2:**
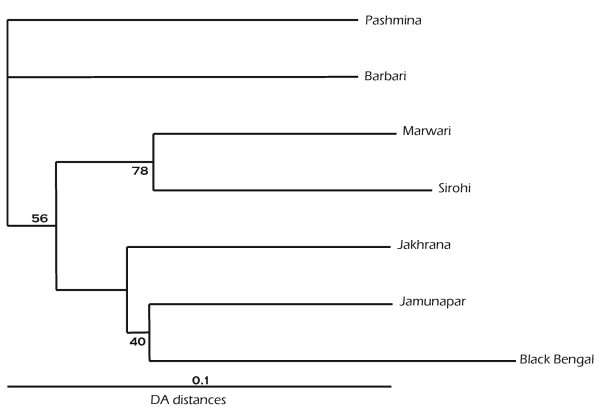
Unrooted NJ tree showing genetic relationship amongst Indian goat breeds. Numbers at the nodes are bootstrapping values from 1000 replicates.

Allele frequencies were used to generate the D_A _genetic distance between each pair of populations and distance matrices were used to build phylogenetic trees using the UPGMA and NJ algorithms. As both trees retained similar structure, only the NJ tree constructed from a matrix of D_A _distances is presented in Figure 2. The D_A _genetic distances and Fst distances between pairs of breeds are shown in Table 5. The lowest distance was observed between Marwari-Sirohi (0.135) and Jamunapari-Jakhrana. The highest distance was observed between Pashmina and BlackBengal and between Barbari and Black Bengal. The NJ tree revealed two different clusters (Fig. 2). The first cluster consisted of the Marwari and Sirohi breeds, and the 2^nd ^cluster consisted of the Jamunapari, Black Bengal and Jakhrana breeds. Bootstrap values ranged from 40 to 78 indicating reliable topology of the phylogeny constructed from D_A _distances. Barbari and Pashmina goats were placed separately in the phylogenetic tree.

**Table 5:** Nei's D_A _genetic distance matrix and Pairwise Fst distance between seven Indian goat breeds (Fst above diagonal and D_A _distance below diagonal)

	**Barbari**	**Jamunapari**	**Black Bengal**	**Jakhrana**	**Marwari**	**Sirohi**	**Pashmina**
**Barbari**		0.064	0.088	0.084	0.075	0.081	0.074
**Jamunapari**	0.185		0.048	0.045	0.048	0.059	0.058
**Black Bengal**	0.221	0.158		0.052	0.053	0.059	0.080
**Jakhrana**	0.190	0.141	0.167		0.053	0.049	0.067
**Marwari**	0.198	0.173	0.214	0.184		0.036	0.061
**Sirohi**	0.215	0.189	0.206	0.182	0.136		0.067
**Pashmina**	0.182	0.184	0.246	0.186	0.176	0.198	

#### Principal component analysis

##### Single-marker analyses

**Figure 3:**
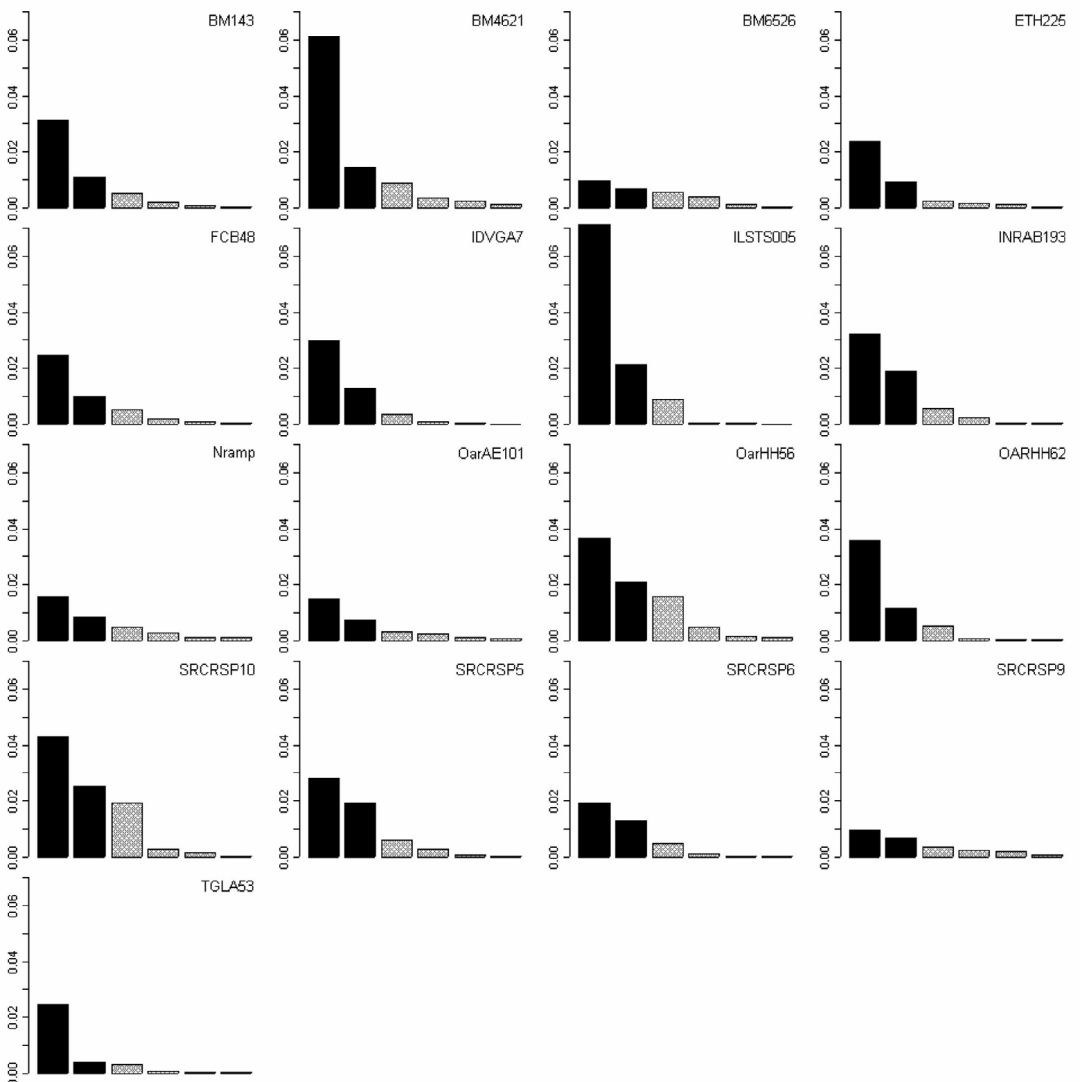
Scree plots of the single-marker PCA.

PCA was performed for each of the 17 markers. Corresponding scree plots are presented in Figure 3. Splitting up the inertia according to axes leads to the so-called scree plot. A scree plot is a simple line segment plot that shows the fraction of total variance in the data as explained or represented by each Principal Component. Contribution of a marker to the construction of an axis is measured by the part of the inertia of this axis that is supplied by the marker. Splitting up the inertia of an axis according to markers enables one to evaluate the degree of consensus of this axis. The inertia was different according to the markers. The most important markers were ILSTS005, BM4621 and SRCRSP10, while, the markers Nramp, OARAE101 and SRCRSP9 did not contribute significantly to inertia. For each single-marker analysis, distances among breeds were computed. Distances were unequal among populations (Kruskall-Wallis test, χ^2 ^= 29.45, 20 d.f, P = 0.08), indicating the existence of a multivariate compromise structure.

##### Global Analysis

**Figure 4:**
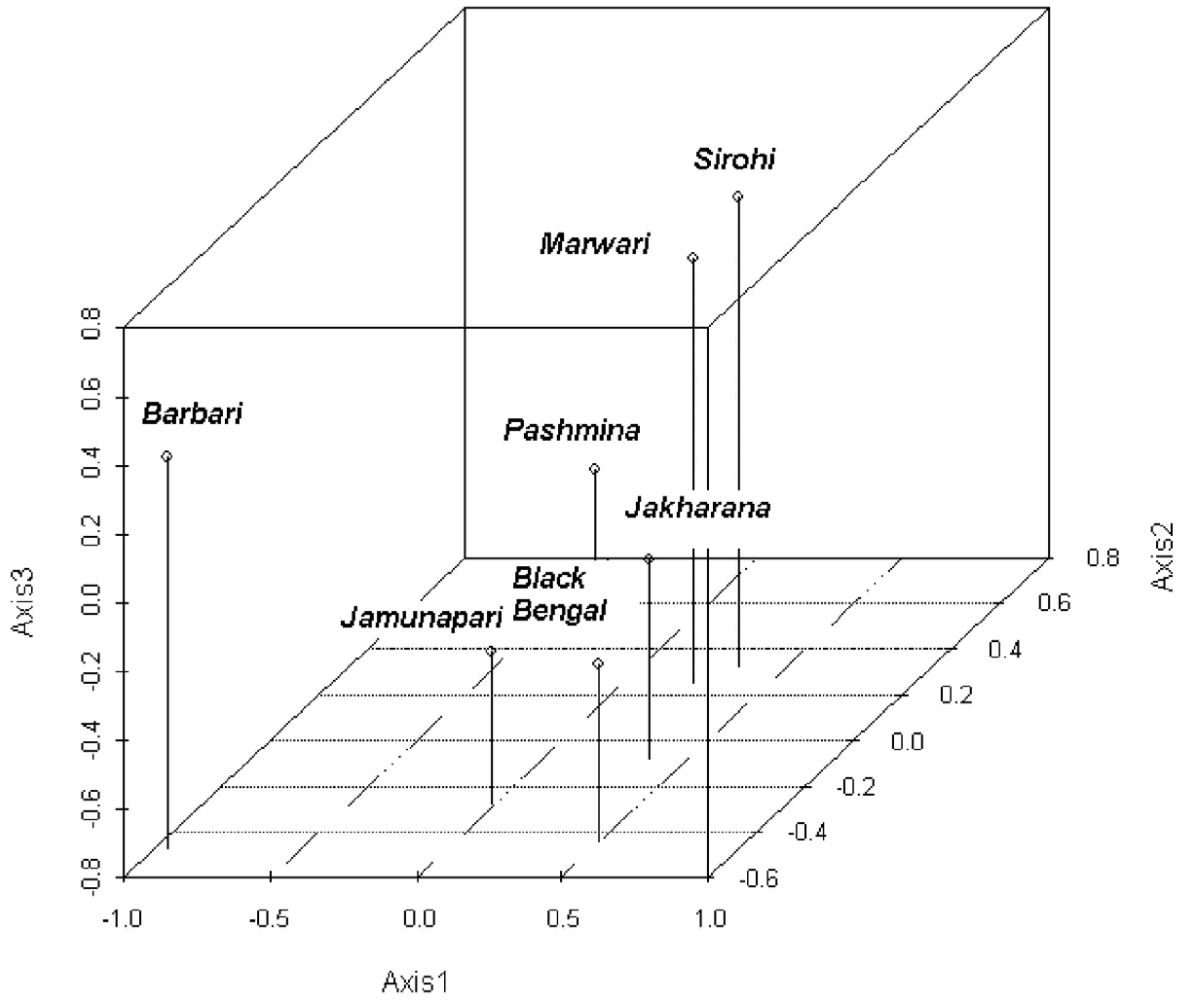
Global Principal Component Analysis (First three principal components).

PCA was performed using the frequencies of the 242 alleles of the 17 markers. The first three principal components explained 65% of the total variation. The global principal component analysis for the first three principal components is presented in Figure 4. The first axis contributed about 27% of the inertia and distinguished the Pashmina and Barbari populations from the other populations, especially the Black Bengal. The second axis contributed 20% of the inertia and separated a cluster containing Barbari, Jamunapari, Jakhrana, and Black Bengal, from a cluster containing Pashmina, Sirohi and Marwari. The third axis contributed about 18% of the inertia and again distinguished a cluster containing Sirohi and Marwari from the breeds Jamunapari, Jakhrana and Black Bengal, but differs from the second axis by the different positions of Barbari and Pahsmina. As a result, these three axes revealed a pattern of association that supports a partition of populations into 4 discrete groups: (1) Barbari, (2) Pashmina, (3) Jamunapari, Jakhrana and Black Bengal and (4) Sirohi and Marwari (Figure 3)

##### Contributions of markers to the global analysis

**Figure 5:**
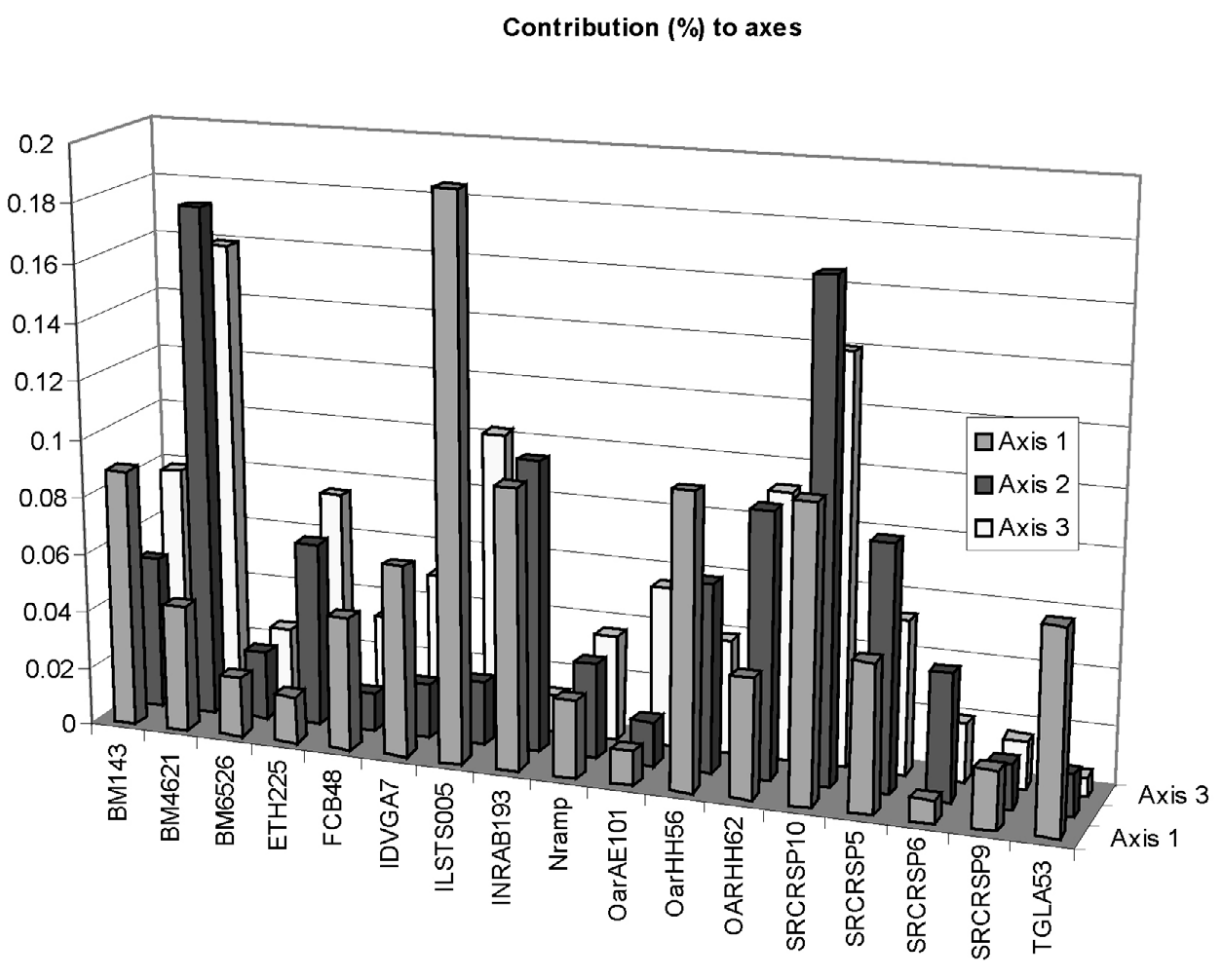
Contribution of markers to the construction of the axes of the global analysis.

The contributions of markers to the construction of axes are plotted in Figure 5. Contributions to axes are variable for all the three axes. Not surprisingly, markers with the greatest inertia contributed the most to the construction of axes: ILSTS005 contributed 20% to the construction of first axis, while BM4621 and SRCRSP10 together contributed 35% (axis 2) and 30% (axis 3). Due to the importance of these three markers, it may be interesting to detail their contributions. Contributions of SRCRSP10 to the three axes were roughly comparable. The corresponding PCA plot indicates that this single-marker tyology shared the most features with the global one, i.e. the separation between Pashmina and Barbari and the separation between these two populations and the others.

On the other hand, BM4621 and ILST005 participated in the construction of only one or two axes. ILSTS005 participated in the construction of the first axis. The corresponding PCA plot indicates that Pashmina and Barbari breeds were isolated from some other populations, as in the fist axis of the global analysis, but the clusters Jakhrana, Jamunapari, Black Bengal and Sirohi, Marwari were not exhibited by this marker. On the other hand, BM4621, which contributes to the construction of the second and third axes, exhibited these clusters, but did not isolate the Barbari and Pashmina breeds. BM4621 revealed three clusters (Pashmina, Barbari, other breeds) in contrast to the four clusters exhibited by the global analysis (Pashmina, Barbari, Black Bengal and Jamnuapari, Jakhrana).

## Discussion

### Breed differentiation

Our genetic analysis of seven Indian goat breeds with 17 microsatellite markers showed higher gene diversity as compared to European and Asian goat breeds. Barker, 1994 [13] and Takezaki and Nei, 1996 [14] suggested that microsatellite loci for genetic diversity studies should have more than four alleles in order to reduce the standard error estimates of genetic distance. The total number of alleles per locus in the present study ranged from 9 to 22. This higher number of alleles for each locus suggested that all the markers used were appropriate to analyse diversity in Indian goats. The mean number of alleles observed over a range of loci in different populations was considered to be a reasonable indicator of genetic variation within the populations [3]. A more appropriate measure of genetic variation within a population was gene diversity (average expected heterozygosity) [15]. Gene diversity for each breed ranged from 0.724 in Barbari to 0.783 in Jakhrana. Takezaki and Nei, 1996 [14] determined that for markers to be useful for measuring genetic variation, they should have an average heterozygosity ranging from 0.3 to 0.8 in the populations. This again confirmed that these markers were appropriate for measuring genetic variation. By analysing mitochondrial HVR1 region in our previous study [12], the lowest haplotype diversity was observed in Pashmina goats (0.926) and the highest haplotype diversity was observed in Jamunapari goats. Microsatellite analysis also revealed that the Pashmina goats exhibited the lowest diversity as compared to other breeds in the present study. The measures of population differentiation indicated variability within breeds and the exact test for population differentiation indicated significant differences between breeds. We observed a large, significant difference between expected and observed heterozygosities in all the Indian goat breeds. This large difference indicates that there is a considerable degree of genetic subdivision within breeds. In India goats are exploited very little by artificial selection, but mtDNA analysis has established new lineages in Indian goats as compared to other goat breeds of the world [12].

As no systematic effort has been made to create distinct goat breeds in India, founder effects and genetic drift may have played major role in differentiation of Indian goat breeds. Population subdivision in Indian goats is also supported by the average proportion of genetic differentiation among breeds (8.03%). As R_ST _values shows the fraction of total variance of allele size between populations, the estimated R_ST _was more than twice the size of G_ST _and F_ST_, suggesting that goat breeds differ in both allele frequency and allele size. In addition, AMOVA indicated that 6.59% of the total genetic variation is between breeds of goats, confirming higher within population diversity in the Indian subcontinent. Mitochondrial DNA analysis in Indian goats showed 83% of variation within breeds and 17% among breeds [12]. The between breed variation in Swiss goats was 17% [8] using microsatellites. Similarly mtDNA analysis showed about 10.7% variation among the goat breeds from Africa, Middle East, Asia and Europe [16].

### Genetic relationship between Indian goat breeds

Takezaki and Nei, 1996 [14] have shown that the construction of a phylogenetic tree depends on the type of population and number of markers used to analyse the population. It has been also showed that increasing the number of loci does not necessarily enhance the reliability of the phylogeny [6]. Takezaki and Nei, 1996 [14] have demonstrated that D_A _and D_C _are the most efficient means of obtaining a correct tree topology on the basis of microsatellite analysis when within population variation is high and distances between each pair of populations are used to build a NJ tree.

The genetic data revealed that the smallest distance is between Marwari and Sirohi and the largest distances are between Pashmina and Black Bengal, Barbari and Black Bengal, and Barbari and Jakhrana. The highest geographical distance between Black Bengal and Pashmina corresponds to the highest genetic distance. The phylogenetic analysis indicated that breeds were grouped according to their geographic locations except Barbari goats. A similar observation of population clustering according to their geographic origin has been reported in cattle [4]. This shows that geographically adjacent populations are more genetically related. The principal component analysis supported the grouping of animals and the distance between breeds was significant. However Barbari goats showed a deviation as they did not cluster with geographically close breeds such as Sirohi, Jamunapari and Jakhrana. The Barbari did not group with any one of the neighboring breeds, consistent with variation in morphological characteristic (ear pattern) and presence of two new lineages by mtDNA analysis. Moreover Jamunapari, Jakhrana and Black Bengal clustered in one group, indicating a shared gene pool, motivating further analysis to establish their migration and origin through a coastal route.

Breeds cluster according to their geographic location. Similar population clustering according to geographic location was previously observed in microsatellite analyses of humans [17], cattle [4] and chickens [18]. Mitochondrial DNA analysis in goats also indicated geographical clustering in the breeds [12]. The result indicated that geographically adjacent populations were more genetically related, perhaps due to founder effects and interbreeding near bordering areas. PCA revealed separation of breeds more clearly between populations of different geographical locations. Some breeds which showed a close phylogenetic relationship are separated more clearly by the PCA plot. Mt-DNA markers are extremely informative for predicting the conformation of the gene pool from maternal inheritance. The Jamunapari is a breed of semi arid regions and isolated in small pocket of Chambal ravines [2]. The Barbari is a goat breed of semi arid regions and distributed over a wide breeding tract. Pashmina goats are found in high altitude Himalayan regions and known for fibre quality. Similarly the Black Bengal is the most prolific breed from the eastern part of India and distributed over a wide area. The Jakhrana is also from semiarid regions of Rajasthan and adapted to a specific locality. The Marwari and Sirohi are breeds of arid regions of western India and distributed over a large breeding tract. The differential existence of breeds varying from hot humid to hot arid, hot humid to cold humid and isolated to particular regions is illustrated by breeds such as the Jamunapari and Jakhrana. PCA isolated the Barbari from other breeds indicating their differential origin, consistent with ear characteristics and existence of two new lineages by mtDNA analysis. Principal component analysis showed clustering of goats according to their geographical origin. Although the breeding tracts of goats are overlapping and no strict breeding policy is adopted to maintain standard breeds in the region, they still maintain genetic distinctness in their natural habitat. Therefore it is necessary to combine genetic data with geographical positioning and to assess the genetic relationship by geostatistical models in further studies.

## Conclusion

In conclusion, this analysis showed that microsatellites as well as mtDNA analysis can be used to classify Indian goat populations into distinct genetic groups or breeds. Phylogenetic and principal component analysis showed the clustering of goats according to their geographical origin. Although the breeding tracts of goats are overlapping and they are spread over all the parts of the country, they still maintain genetic distinctness in their natural habitat.

## Methods

### Genetic Stocks

A total of 302 goats representing 7 major breeds of India were sampled from their natural habitat. The breeds studied have been grouped into 5 different types based on their utility and size (Table 1). Summaries of goat breeds sampled from their natural habitat are described in Table 1.

### Sample collections and DNA isolation

About 10 ml of blood samples were collected from each animal's jugular vein using EDTA vacutainers and stored at 4°C until DNA isolation. An effort was made to collect samples from unrelated individuals based on information provided by farmers. The geographical distribution of the breeds from different regions sampled for the study is described in Table 1. DNA was isolated from blood as described elsewhere [19].

### Selection of STR markers

The markers were chosen from the existing bovine, ovine and caprine genetic maps [20-22] with an effort to cover all chromosomes having high heterozygosity. Twenty-three STR markers were included in this study for analyzing the variation among various goat breeds (Table 2). All the DNA samples were analysed with 23 STR markers. Each 10μl PCR reaction mixture consists of 10 ng of template DNA, 1× buffer, 200 μM dNTPs, 2.5 mM MgCl_2_, 1 U of AmpliTaq Gold (Perkin Elmer) and 10 pM of each primer. Amplification conditions for these markers were as follows: Initial denaturation at 95°C for 10 min. followed by 95°C for 1 min, specific annealing temperature for each marker as given in Table 2 and 30 s at 72°C for 30 cycles. An final extension temperature of 72°C for 5 min was used for each reaction.

### GeneScan and genotyping

One micro liter of PCR amplicons and 0.5 μl of size standard (GS-ROX500) were mixed with 2.5 μl of loading dye (formamide: bluedextrin; 5:1), denatured (94°C for 2 mins) and electrophoresed in 5% Long Ranger (FMC) gel, using ABI 377 automated DNA sequencer (Perkin Elmer). GeneScan (Perkin-Elmer) software was used to analyse the gel image and Genotyping software (Perkin-Elmer) was used to get the allele size of each amplicons.

### Statistical analysis

Exact tests for deviations from Hardy-Weinberg equilibrium (HWE) were performed by the GENEPOP Package [23]. The program performs a probability test using a Markov Chain (dememorization 10,000, batches 100, iteration per batch 1000). Genetic disequilibrium was estimated between all locus pair using GENEPOP. The mean number of alleles per locus (NA), total number of alleles (TNA), the observed heterozygosity (Ho) and the expected heterozygosity (He) under HWE were computed using FSTAT [24] and AGArst software [25]. To test sample bias, Allelic richness based on minimum sample size (n = 31) was estimated using FSTAT [24]. Significance levels of difference in NA and He between populations were tested using a Wilcoxon's signed ranks test.

Analysis of molecular variance (AMOVA), Fst and pairwise difference were computed using ARLEQUIN ver 3.11 [26,27]. A second estimator of gene differentiation, R_ST_, was calculated which accounts for variance in allele size and was defined for genetic markers undergoing a stepwise mutation model. The D_A _genetic distance [28] was computed with DISPAN [29] to establish genetic distance between populations. A NJ/UPGMA tree was constructed in comparison for Indian goat breeds using the PHYLIP Package Ver3.6 [30]. The significance of population difference was tested using the exact test of population differentiation proposed in GENEPOP software based on allele frequency variation.

### Principal Component Analysis (PCA)

As admixture in the breeding tract of goats is very common and some populations are known to be admixed, we used a multivariate procedure to represent population relationships. Multivariate procedures are recommended in this situation because the admixed populations are not original evolutionary units and may be misrepresented by phylogeny-based tree-building techniques [31]. Prior to multivariate analysis, we tested for congruence of loci following the two-step procedure developed by Moazami-Goudarzi and Laloe, 2002 [5]. This is because a consensus representation of population relationships is not meaningful if single markers are not congruent, as would occur if many of the distances among populations based on individual loci were negatively correlated [4]. Euclidean distance matrices between all populations were first generated for each locus. Then, a Kruskall-Wallis test [32] on rank scores of standardized distances between populations was carried out and a significant Kruskal-Wallis test indicated that a compromise structure exists because distances are unequal between populations. If a compromise structure exists, then a multivariate analysis, such as principal components analysis will be meaningful. PCA was then done using the allelic frequencies as variables. It leads to a representation of populations as a cloud of points in a metric space. Comparison between the inertia of single-marker enables to compare the typological value of the markers. Inertia can be split up according to axes and/or loci [33]. All computations were done using the R software [34]. More specifically, computations relative to PCA were done with the ade4 package [35].

## Authors' contributions

PKR, LS and KT conceived and designed the experiments. MBJ and AM performed the experiments. PKR, MBJ, AM and DL performed the data analysis. PKR, MBJ and KT wrote the manuscript. All authors read, revised and approved the final manuscript.

## References

[B1] Acharya RM (1982). Sheep and Goat breeds of India, Animal Production and Health.

[B2] Rout PK, Mandal A, Singh MK, Roy R, Sharma N, Haenlein GFW (2004). Jamunapari - a diary goat breed in India. Dairy Goat Journal (USA).

[B3] Cavalli-Sforza LL (1998). The DNA revolution in population genetics. Trends Genet.

[B4] MacHugh DE, Shriver MD, Loftus RT, Cunningham P, Bradley DG (1997). Microsatellite DNA Variation and the evolution, domestication and phylogeography of taurine and zebu cattle (Bos Taurus and Bos indicus). Genetics.

[B5] Moazami-Goudarzi K, Laloe D (2002). Is a multivariate representation of genetic relationships among populations always meaningful?. Genetics.

[B6] Litt M, Luty JA (1989). A hypervariable microsatellite revealed by *in vitro *amplification of a dinucleotide repeat within the cardiac muscle actin gene. Am J Hum Genet.

[B7] Buchaman FC, Adams LJ, Littlejohn RP, Maddox JF, Crawford AM (1994). Determination of evolutionary relationships among sheep breeds using microsatellites. Genomics.

[B8] MacHugh DE, Loftus RT, Cunningham P, Bradley DG (1998). Genetic structure of seven European cattle breeds assessed using 20 microsatellite markers. Animal Genet.

[B9] Saitbekova N, Gaillard C, Obexer-Ruff G, Dolf G (1999). Genetic diversity in Swiss goat breeds based on microsatellite analysis. Anim Genet.

[B10] Yang IS, Zhao H, LI K, Peng ZZ, Montgomery GW (1999). Determination of genetic relationships among five indigenous Chinese goat breeds with six microsatellite markers. Anim Genet.

[B11] Mateus JC, Penedo MCT, Alves VC, Ramos M, Rangel-Figueiredo T (2004). Genetic diversity and differentiation in Portuguese cattle breeds using microsatellites. Anim Genet.

[B12] Joshi MB, Rout PK, Mandal A, Singh L, Thangaraj K (2004). Phylogeography and origin of Indian domestic goats. Mol Biol Evol.

[B13] Barker JSF (1994). A global protocol for determining genetic distances among domestic livestock breeds. Proceedings of the 5th world congress of Genetics Applied to Livestock Production.

[B14] Takezaki N, Nei M (1996). Genetic distances and reconstruction of phylogenetic trees from microsatellite DNA. Genetics.

[B15] Nei M (1987).

[B16] Luikart G, Gielly L, Excoffier L, Vigne J, Bourvet J, Taberlet P (2001). Multiple material origins and weak phylogeographic structure in domestic goats. Proc Natl Acad Sci (USA).

[B17] Bowcock AM, Ruiz-Linares A, Tomfohrde J, Minch E, Kidd JR, Cavalli-Sforz LL (1994). High resolution of human evolutionary trees with polymorphic microatellites. Nature.

[B18] Wimmers K, Ponsuksili S, Hardge T, Valle-Zarate A, Mathur PK, Horst P (2000). Genetic distinctness of African. Asian and South American local chickens. Anim Genet.

[B19] Thangaraj K, Joshi MB, Reddy AG, Gupta NJ, Chakraborthy BN, Singh L (2002). CAG repeat expansion in androgen receptor gene is not associated with male infertility in Indian populations. J Androl.

[B20] Barendse W, Armitage SM, Kossarek LM, Shalom A, Kirkpatrick BW, Ryan AM, Clayton D, Li L, Neibergs HL, Zhang N (1994). A genetic linkage map of the bovine genome. Nat Genet.

[B21] Kemp SJ, Hishida O, Wambugu J, Rink A, Longeri ML, Ma RZ, Da Y, Lewin HA, Barendse W, Teale AJ (1995). A panel of polymorphic bovine, ovine and caprine microsatellite markers. Anim Genet.

[B22] Vaiman D, Koutta O, Oustry A, Elsen JM, Manfredi E (1996). Genetic mapping of the autosomal region involved in XX sex-reversal and horn development in goats. Mamm Genome.

[B23] Raymond M, Rousset F (1995). (GENEPOP) (version 3.1d): population genetics software for extract test and ecumenicism. J Hered.

[B24] Goudet J (1995). FSTAT (version 1.2): a computer program to calculate F-statistics. J Hered.

[B25] Harley EH (2002). AGAR_ST_, version 2.8, a program for calculating allele frequencies, G_ST _and R_ST _from microsatellite data.

[B26] Excoffier L, Smouse PE, Quattiro JM (1992). Analysis of molecular variance inferred from metric distances among DNA haplotypes: application to human mitochondrial DNA restriction data. Genetics.

[B27] ARLEQUIN ver 3.11. http://cmpg.unibe.ch/software/arlequin3/.

[B28] Nei M, Tajima F, Tateno Y (1983). Accuracy of estimated phylogenetic trees from molecular data. II. Gene frequency data. J Mol Evol.

[B29] Ota T (1993). DISPAN. Genetic Distance and Phylogenetic Analysis. Pennsylvania State University.

[B30] Felsenstein J (1993). PHYLIP (phylogeny inference package). Versions 3.5c.

[B31] Cavalli-Sforza LL, Menozzi P, Piazza A (1994). The history and geography of human genes.

[B32] Hollander M, Wolfe DA (1973). Non parametric statistical inference.

[B33] Laloë D, Moazami-Goudarzi K, Chessel D (2002). Contribution of individual markers to the analysis of the relationships among breeds by correspondence analysis. 7th World Congress on Genetics Applied to Livestock Production, August 19–23, Montpellier, France Communication 26-06.

[B34] Ihaka R, Gentleman R (1996). R: A language for data analysis and graphics. J Comput Graph Sta.

[B35] Chessel D, Dufour AB, Thioulouse J (2004). The ade4 package. I – One table methods. The R newsletter.

